# WRKY Proteins: Signaling and Regulation of Expression during Abiotic Stress Responses

**DOI:** 10.1155/2015/807560

**Published:** 2015-03-24

**Authors:** Aditya Banerjee, Aryadeep Roychoudhury

**Affiliations:** Postgraduate Department of Biotechnology, St. Xavier's College (Autonomous), 30 Mother Teresa Sarani, Kolkata, West Bengal 700016, India

## Abstract

WRKY proteins are emerging players in plant signaling and have been thoroughly reported to play important roles in plants under biotic stress like pathogen attack. However, recent advances in this field do reveal the enormous significance of these proteins in eliciting responses induced by abiotic stresses. WRKY proteins act as major transcription factors, either as positive or negative regulators. Specific WRKY factors which help in the expression of a cluster of stress-responsive genes are being targeted and genetically modified to induce improved abiotic stress tolerance in plants. The knowledge regarding the signaling cascade leading to the activation of the WRKY proteins, their interaction with other proteins of the signaling pathway, and the downstream genes activated by them are altogether vital for justified targeting of the *WRKY* genes. WRKY proteins have also been considered to generate tolerance against multiple abiotic stresses with possible roles in mediating a cross talk between abiotic and biotic stress responses. In this review, we have reckoned the diverse signaling pattern and biological functions of WRKY proteins throughout the plant kingdom along with the growing prospects in this field of research.

## 1. Introduction

All sustaining living organisms especially the sessile ones like plants are always exposed to a variety of conditions which may cause deleterious impacts on all phenological stages of development. Such adverse conditions are called stress for that particular organism. Harsh environmental conditions which hinder the proper physiological growth of the plant system are called abiotic stresses which include drought, soil salinity, heavy metal, low temperature, radiation, and other forms of oxidative stresses. Adaptation to such environmental stress is essential not only for development of the individual plant, but also for the stability of its successive generations. Genetic manipulation of crop plants has been undertaken to develop stress-resistant crop varieties. The notion of bringing up such genetically modified (GM) crops has become stronger after it was estimated that the maximum worldwide crop yield loss (70%) can be attributed to abiotic stress sensitivity of the crops.

Natural evolution in plants has also enhanced multiple level molecular mechanisms to tackle abiotic stress by the induction of stress-responsive and stress-tolerance genes [1]. Such induction is highly dependent on proper perception and transduction of the environmental cues via a signaling cascade [2]. Transcriptional regulation of the stress-induced genes plays a pivotal role in developing stress tolerance in plants. Such regulation is mainly dependent on the temporal and spatial functioning of the transcription factors (TFs) [3]. Presence of 2100 and 2300 TFs has been reported in* Arabidopsis thaliana* and* Oryza sativa*, respectively. This shows the enormous importance of TFs in regulating gene expression; otherwise, such a large portion of the genome would not have been devoted for coding the TFs alone [4]. Upregulation of specific TFs corresponding to that of some stress-induced genes has unleashed a complex network of interconnected cross talks. Researchers are trying to find whether specific stress-responsive TFs, apart from upregulating their target genes, also essentially regulate a complete package of stress-induced responses like posttranslational and epigenetic modifications, namely, variable nucleosome distribution, histone modification, DNA methylation, and synthesis of nonprotein-coding RNAs (npcRNAs) [5].

## 2. TFs in Abiotic Stress

TFs have been a major target in improving stress tolerance in plants due to their ability to control critical downstream responses by regulating target gene transcription. Entire cascades of signal transduction get activated when TFs interact with specific* cis*-acting response elements in the promoters of stress-inducible genes. This enhances combinatorial tolerance against multiple stresses [6]. Thus, these TFs themselves can be genetically upregulated to increase the stress-induced gene responsiveness and hence the overall stress tolerance. The principal TFs involved in stress management lie in the AP2/ERF (Apetala2/ethylene response factor), bZIP (basic leucine zipper), NAC (No Apical Meristem, ATAF1/2, Cup-Shaped Cotyledon 2), MYB, C2H2 Zn finger, SBP (Squamosa-Promoter Binding Protein), and WRKY (TFs containing highly conserved WRKY domain) superfamilies [7]. The bZIP TF family consists of a large number of TFs, with diverse roles and ability to bind to abscisic acid- (ABA-) responsive elements (ABREs) [8]. The MYB family of TFs are regulators of several responses pertaining to secondary metabolism, cell cycle, biotic defence, and abiotic stress [9]. The NAC TFs like* stress responsive NAC* (*SNAC*) genes when overexpressed improve the drought tolerance in rice [10]. In this review, we will focus on the intricate relation of one of the largest families, that is, the WRKY superfamily of TFs, in enhancing stress tolerance in various plant species.

## 3. WRKY Proteins: Structural Characterization and Classification

The WRKY TFs were first identified in sweet potato (SPF1) as DNA-binding proteins [11]. Evolution of Mutator or Mutator-like transposable elements (MULEs) gave rise to the WRKY-GCM1 superfamily of Zn finger TFs [12]. The TFs containing the DNA binding domain, GCM, have been clumped together with the WRKY TFs to be classified as the WRKY-GCM1 superfamily [13].* WRKY* genes are quite common in plants, though some nonplant species have also been identified which carry such genes in their genomes. 74* WRKY* genes in* Arabidopsis*, more than 100 genes in rice, 197 genes in soybean, 66 genes in papaya, 104 genes in poplar, 68 genes in sorghum, 38 genes in* Physcomitrella patens*, 35 genes in* Selaginella moellendorffii*, 80 genes in* Pinus*, more than 45 genes in barley, 56 genes in* Ricinus communis*, 119 genes in the B73 inbred line of maize, 55 genes in* Cucumis sativus*, 120 genes in* Gossypium raimondii*, and 59 candidate genes in* Vitis vinifera* have been identified [14–19]. The large number of genes present in the genomes of several plant species does hint about a pivotal role played by these TFs in downstream gene activation. The WRKY domain has a conserved N-terminal sequence of WRKYGQK along with a Zn finger-like motif, which can be either Cx_4-5_Cx_22-23_HxH (C2H2 type) or Cx_7_Cx_23_HxC (C2HC type). The WRKY domain can be 60 amino acids long and binds DNA [20]. Slight variations of WRKYGQK can be found in some WRKY TFs. The WRKY DNA-binding domain generally binds to the W-box elements containing the TTGAC(C/T) motif, though the flanking sequence adjoining the W-box dictates the binding selectivity of the TF [20]. For example, in contrast to other WRKY TFs, WRKY6 and WRKY11 of* Arabidopsis* have high affinity towards a G base upstream of the core motif of the W-box [21].

*Arabidopsis* being the model plant has the most well classified list of WRKY proteins (Table 1). These proteins have been divided into three distinct groups depending on the numbers of WRKY domains present and the diversity in the Zn finger motifs found in them [22]. The bifurcation in the groups occurred mainly due to the number of WRKY domains. The proteins belonging to Group I have two distinct WRKY domains, while the proteins belonging to both Groups II and III have single domains. The difference between the proteins of Groups II and III lies in the fact that the former group consists of proteins with the same Cys2-His2 Zn finger motif, while those belonging to the latter group have different Cys2-His/Cys Cys2-His2 Zn finger motif [14]. On the basis of the presence of additional conserved structural motifs, Groups II and III have been further divided into subgroups. Group IV WRKY has been characterized by the loss of the Zn finger motif. Though these proteins were thought to be nonfunctional, they were found in higher algae (*Bathycoccus prasinos*) and some plants like rice and* Vitis vinifera*. Recently, the VvWRKYs from* V. vinifera* have been reported to play crucial roles in generating cold stress tolerance in grapevines (Table 1). Structures like nuclear localization signal (NLS), leucine zippers, Ser/Thr rich stretches, Gln and Pro rich stretches, kinase domains, and pathogenesis-related TIR-NBS-LRR domains have also been identified in the WRKY proteins which infers the diverse roles played by these proteins in multifarious signaling cascades [14].

## 4. WRKY Proteins in Stress

Under normal cellular conditions, the* WRKY* genes regulate important functions related to the developmental processes in plants. The expression of the* Dactylis glomerata WRKY* gene,* DGE1*, is essential for proper somatic embryogenesis [23]. Strong expression of* ScWRKY1* is induced in the fertilized ovules in potato at the late torpedo stage of embryogenesis [24]. Transparent Testa Glabra 2 (TTG2) is also named as WRKY44 and is involved in the regulation of development of trichomes and root hairs [25]. Starch production in endosperm is governed by a WRKY protein, SUSIBA2. High expression of* Miniseed3* gene encoding AtWRKY10 has been reported in pollens, globular embryos, and developing endosperms [25].

The WRKY proteins play prominent roles in the regulation of transcriptional reprogramming associated with plant stress responses (Table 2). Such WRKY-mediated transcriptional response can be against both biotic and abiotic factors [14]. The WRKY proteins function via protein-protein interactions and even cross regulation and autoregulation. Detailed study on the mechanisms of signaling and transcriptional regulations has unveiled the association of the WRKY proteins with mitogen-activated protein kinases (MAPKs), MAPKKs, 14-3-3 proteins, calmodulin, histone deacetylases, disease-resistance proteins, and other WRKY TFs [26].

Though we will focus on the roles of WRKY in abiotic stress, these proteins have diverse roles in biotic stress as well. Disease resistance and crop yield in the important tropic crop* Theobroma cacao* have been developed by identifying specific WRKY loci as the genetic markers [27]. In this review, efforts have been made to improve WRKY loci as genetic markers against both abiotic and biotic stresses. Upon designing the complementary PCR primers against the WRKY domains of Group I and Group II (a–c) proteins, 16 WRKY fragments were isolated from a mixture of* T. cacao* DNA using one pair of primers [27]. Four among these 16 fragments contained single nucleotide polymorphisms within the intron and could be considered as molecular markers after further experimentations [27].

Abiotic stresses like drought, salinity, radiation, and cold induce the activity of several WRKY proteins which function in synchronization to confer resistance against the particular stress or provide a combinatorial effect on multiple stress resistance. Out of the 13* OsWRKY* genes, 11 show variable responses towards salt stress, polyethylene glycol (PEG), cold or heat stresses [28]. In wheat, majority (8 out of 15) of the* WRKY* genes were transcribed in response to cold, heat, NaCl, and PEG treatment [29]. Induction of 18* AtWRKY* genes in the roots of* Arabidopsis* plants treated with 150 mM NaCl was confirmed via microarray profiling [30]. The rapid expression of* WRKY* genes following stress has led to the argument whether such transient increase in WRKY proteins is independent of the* de novo* synthesis of the regulatory factors [22]. Both activation of adaptive responses and transcriptional regulation of stress-induced genes are actually possible due to the immediate early expression of* WRKY* genes. Thus, the WRKY protein level in the cell increases sharply and this rise in protein accumulation aids them to regulate target gene transcription by associating with the* cis*-acting response elements [14]. Table 2 is a concise representation of the role of* WRKY* genes in stress. However, the table does emphasise on the fact that WRKY TFs mediate tolerance to several abiotic stresses, via transcriptional reprogramming and control of signaling cascades. Expression patterns of WRKY TFs thus have been intricately studied in order to find a proper basis and clue towards overexpressing particular WRKY proteins of choice through transgenic approach.

## 5. WRKY-Dependent Signaling Pathways in Abiotic Stress

### 5.1. Autoregulation and Cross Regulation

The notion of WRKY proteins involved in critical stress responses obviously makes extensive regulation of the signaling pathway mandatory. In response to both external and internal stimuli, WRKY proteins bind to W-box-containing promoters and trigger the expression of target stress-responsive genes. This triggering is often autoregulated by the WRKY protein itself or by separate WRKY TFs (cross regulation) [26]. Three Group IIa WRKY proteins in* Arabidopsis*, AtWRKY18, AtWRKY40, and AtWRKY60, have leucine zipper motifs at their N-termini via which they interact among themselves [31]. The PcWRKY1 of parsley (*Petroselinum crispum*), apart from binding to its target W-box, also has affinity towards binding the promoters of* PcWRKY3* and even that of the marker gene PcPR1 [32]. The MAPK3/6 activates* WRKY33* expression. The WRKY33 proteins* in vivo* autoregulate their expressions, via a positive feedback loop by binding to their own promoter [33]. Cross regulation among WRKY25, WRKY26, and WRKY33 is essential in promoting tolerance against high temperature stress [34]. The AtWRKY18, AtWRKY40, and AtWRKY60 are directly bound to their respective promoters in order to negatively regulate their expression patterns [13]. The above instances obviously prove the importance of autoregulation and cross regulation in maintaining the homeostasis of WRKY protein expression in the cell.

### 5.2. Regulation of* WRKY* Expression by MAPKs

**Figure 1:**
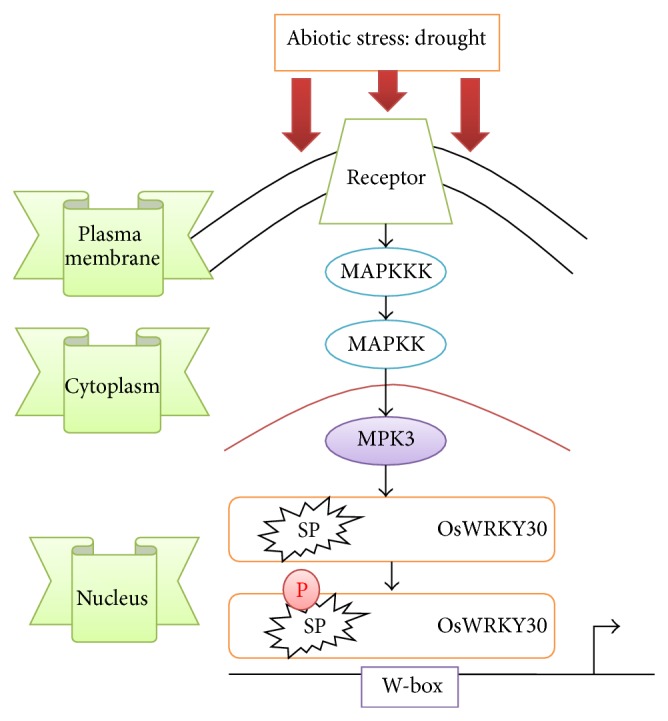
The mitogen-activated protein kinase (MAPK) pathway induces the activity of OsWRKY30 during drought stress. Stress signals are sensed via a transmembrane receptor, which with the help of some unknown molecules and adaptor proteins activates the MPK/MAPK pathway. This leads to the phosphorylation and activation of the MPK3. MPK3 phosphorylates the target Ser residue in the SP motif of OsWRKY30 and activates the same. The activated WRKY protein then undergoes a conformational change which favourably allows it to bind to the W-box of its target gene to induce transcription. The protein product encoded by the target gene probably helps the plant system in combating the drought stress.

The MAPKs play important roles in transduction of downstream signals in ABA-dependent stress response. Wound-induced protein kinase (WIPK) and salicylic acid- (SA-) induced protein kinase (SIPK) play important roles in biotic stresses like pathogen invasions [35]. The AtMPK3, AtMPK6, and AtMPK4 are activated during both abiotic and biotic stresses [36]. The MAPK cascades phosphorylated OsWRKY30 which enhanced the drought tolerance in rice. Point mutation of Ser in the Ser-Pro (SP) site resulted in a drought-sensitive crop [37]. This illustrates the crucial role played by MAPK phosphorylation in proper OsWRKY30 activity (Figure 1). Recent reports have characterized the presence of two pollen-specific WRKY TFs (WRKY34 and WRKY2) during male gametogenesis in* Arabidopsis thaliana*. Overexpression of* WRKY34* using a strong pollen-specific promoter led to the phosphorylation of the WRKY34 protein by MPK6 and MPK3 [38]. Mutations in the phosphorylation sites in WRKY34 compromised its functions* in vivo* [38].* In vivo* phosphorylations of WRKY TFs by MAPKs have also been recently reported [39]. The MPK3/MPK6 cascade along with the downstream WRKY TF has been depicted to induce ethylene production through regulation of ACC synthase activity [40]. The MAPK cascades involved in phosphorylating WRKYs involved in abiotic stress are less studied in comparison to the biotic counterparts. However, the knowledge of these signaling cues can impose further improvements in designing stress-tolerant transgenic crops.

## 6. Interaction between WRKY TFs and Associated Factors in Abiotic Stress

### 6.1. VQ Proteins

VQ proteins are a group of cofactors containing a short VQ-related motif (FxxxVQxLTG). Out of the 34* VQ* genes reported in* Arabidopsis*, the majority do not contain any intron and encode small proteins of 100–200 amino acid residues having the consensus VQ motif. It has been reported that these 34 VQ proteins can interact with WRKY TFs in yeast [41]. The VQ proteins are often activated by MAPK cascades in response to stress signal cues. During post-association with MAPK substrate1 (MKS1), VQ protein interacts with AtWRKY33 and AtWRKY25 to act as a substrate of MAPK4 [42]. Interaction of VQ proteins with WRKY TFs results in stimulation or inhibition of the latter to bind to its specific DNA. This mainly occurs because VQ protein binding manipulates the WRKY TF to change its preference for the nucleotides flanking the conserved W-box [43]. The resulting alteration in target gene specificity of the WRKY TF gives rise to a changed biological downstream response. Diversification of WRKY TF-induced responses may also result from interactions with multiple VQ proteins [43]. Tolerance to multiple abiotic stresses occurred in* Arabidopsis* when the WRKY33 interacted with multiple VQ proteins including Sigma Factor-Interacting Protein1 (SIB1) and SIB2, via the C-terminal of the WRKY domain. This interaction induced the DNA binding activity of AtWRKY33 [44]. SIB1 and SIB2 have been assumed to play roles in regulation of transcription and retrograde signaling from chloroplast and mitochondria to the nucleus. VQ proteins have also been hypothesised to induce chromatin remodelling as an abiotic stress response [44]. MVQ1 is a VQ-motif-containing protein which was recently depicted to control WRKY-regulated defense gene expression [45].

### 6.2. Histone Modifying Chromatin Remodelling Complex

A condensed chromatin structure wrapped within the nucleosomal complex in association with histone proteins and other packaging factors does not easily interact with the transcriptional machinery. So, when the plant is not under stress, the gene remains transcriptionally silent. The sensing of environmental cues signals the WRKY TFs to induce the transcription of target stress-inducible genes. The packed chromatin structure has to be loosened to facilitate proper association of the transcription complex at the promoter site. Thus, it is often considered that such chromatin remodelling complex, along with autoregulation and cross regulation, plays a crucial role in WRKY TF-induced responses.* AtWRKY70* was reported to be stimulated by* Arabidopsis* homolog of trithorax (ATX1) and, as a result, the nucleosomal histone H3K4 trimethylation occurred [46]. Epigenetic regulation by histone methyltransferase is endowed upon* AtWRKY53*, a senescence regulator. Activation of* AtWRKY53* in response to senescence triggered the rise in H3K4 dimethylation and H3K4 trimethylation at the 5′ end and coding regions of* WRKY53* [47]. In* Musa acuminata*, the protein encoded by the linker histone H1 gene (*H1S1*) interacted with MaWRKY1 in response to the stress caused by the postharvest ripening of fruits induced by ethylene. The induction of* MaH1S1* is also accelerated in presence of jasmonic acid (JA), ABA, and hydrogen peroxide and under chilling stress [48]. Overexpression of* AtWRKY38* and* AtWRKY62* enhanced the resistance of the plants to pathogenic attack by* Pseudomonas syringae*. It has been reported that the proteins encoded by these genes interact with Histone Deacetylase 19 (HD19). Overexpression of HD19 retarded the activities of AtWRKY38 and AtWRKY62 as TFs [49].

### 6.3. Calmodulin and 14-3-3 Proteins

The WRKY proteins have a calmodulin (CaM) binding domain (CaBD). Site directed mutagenesis studies have confirmed the importance of this domain in WRKY TFs to bind CaM [43]. The AtWRKY7 associated with CaM through its own CaBD. The AtWRKY7 CaBD consists of VAVNSFKKVISLLGRSR. Ten other* Arabidopsis* Group IId WRKY proteins have been found to possess such related CaBDs (DxxVxKFKxVISLLxxxR). Thus, these proteins also have a tendency to interact with CaMs [50]. In case of overlapping WRKY-WRKY interactions, the steric hindrance prefers WRKY-CaM interaction, provided that the calcium concentration in the cell is high [43].

Seven WRKY proteins in* Arabidopsis* have been identified via proteomic profiling of tandem affinity-purified 14-3-3 complexes to interact with 14-3-3 proteins [51]. Out of these, AtWRKY6 is induced under phosphate starvations. AtWRKY18 and AtWRKY40, complexed with 14-3-3 proteins, participate in ABA signaling [52]. WRKY proteins interacting with 14-3-3 proteins are subjected to phosphorylation by the latter in response to stress-activated signaling cascades [53]. The 14-3-3 proteins are also capable of dimerization and each dimer binds two substrates. Thus, WRKY proteins with phosphorylated binding sites have the tendency to associate with other factors and proteins by mutual interactions with true 14-3-3 dimers. This is the case of indirect association of WRKY with other proteins in the complex [43, 54]. Further studies are required which will aid in developing the database of dynamic interactions of 14-3-3 proteins with WRKY TFs in the spatiotemporal context of abiotic stress signaling cascades.

## 7. Cross Talk between WRKY TF and ABA-Mediated Signaling

WRKY superfamily of TFs is the major regulator in plant defence and SA-mediated signaling. However, significant instances are there which show that these TFs also participate in ABA-mediated signaling [55]. ABA is the universal stress hormone and the WRKY proteins associated with ABA signaling can definitely influence stress-induced responses. AtWRKY40 has been characterised as a negative regulator of ABA signaling during seed germination. AtWRKY40 also interacts with AtWRKY18 and AtWRKY60 to inhibit the expression of crucial stress-responsive genes [52]. WRKY18 and WRKY60 interact with the W-box of the downstream* ABA Insensitive* genes like* ABI4* and* ABI5* in order to repress their expression [56]. Utilising a stable transgenic reporter or effector system, it was observed that WRKY18 and WRKY60 act as weak transcriptional activators, while WRKY40 is a transcriptional repressor in plant cells [57]. WRKY63 in* Arabidopsis* is responsible for enhanced drought tolerance. Increased ABA sensitivity and reduced drought tolerance were observed when the* WRKY63/ABA Overly Sensitive3* (*ABO3*) was disrupted. The drought tolerance reduced especially due to unresponsive ABA-induced stomatal closure. AtWRKY63 binds to the promoters of* ABA Responsive Element Binding Proteins/Factors* (*AREB1/ABF2*) [13]. Reports on the relation between ABA and abiotic stress-mediated* WRKY* genes have identified 16 ABA-related* WRKY* genes in rice. 12 of them were seen to have higher expression in response to cold, drought, and salinity [58]. A study showed that the cross talk between Gibberellic Acid (GA) and ABA is mediated by OsWRKY51 and OsWRKY71 in rice. The expression of the genes encoding these two WRKY proteins is induced by ABA, and this results in a high ratio of OsWRKY51/OsWRKY71 repressors to GAMYB activator [59]. GA induction of *α*-amylase promoter (*Amy32b*) is suppressed due to the binding of OsWRKY51/OsWRKY71 repressors with the respective W-boxes. In such a situation, GA induces the production of GAMYB and inhibits OsWRKY51 and OsWRKY71. Due to higher levels of GAMYB, the *α-amylase* gene expression increases [59]. Other activators of *α-amylase* gene expression via GA are protein factors like OsDOF3, RAMY, and OsMYBs1/OsMYBS2. The repressors other than those mentioned above are KGM and HRT [59, 60].

*Larrea tridentata* (creosote bush) due to its xerophytic evergreen nature has very high tolerance towards drought. WRKY21 of 314 amino acid residues with localization in the nucleus has been isolated from the creosote bush. LtWRKY21 binds to the promoter of ABA-responsive gene* HVA22* and promotes transcription of the same. This activation of transcription is dependent on the levels of LtWRKY21 [61]. The HVA22 protein has important roles in combating multiple abiotic stresses. High gene expression occurs due to coexpression of activators like* VP1* and* ABA Insensitive5* (*ABI5*), along with* LtWRKY21*. The dominant negative mutant protein phosphatases like abi1-1 are not inhibitors of* LtWRKY21*,* VP1*, and* ABI5* coexpression, though these mutant phosphatases are negative regulators of ABA signaling. This proves the fact that the complex of LtWRKY21, VP1, and ABI5 obviously regulates downstream of ABI1 in ABA-mediated response cascades during abiotic stress [61]. AtWRKY57 has shown enhanced expression in response to higher ABA levels conferring higher drought tolerance to the plant [62]. Chromatin immunoprecipitation (ChIP) assays confirmed the binding of WRKY57 to the W-box of* Responsive to Desiccation 29A* (*RD29A*) and* 9-cis-epoxycarotenoid dioxygenase 3* (*NCED3*) promoters. Thus, WRKY57 functions by directly inducing stress-responsive genes [62]. In rice, OsWRKY45-1 and OsWRKY45-2 participate in ABA-mediated responses during abiotic stress. OsWRKY45-2 had a negative effect on ABA-induced downstream responses to salt stress. These two WRKY proteins have also been proved to regulate ABA-dependent signaling during drought and low temperature stresses [63]. WRKY proteins can act both as activators and repressors to ABA-inducible promoters. OsWRKY24 and OsWRKY45 act as the repressors, while OsWRKY72 and OsWRKY77 act as the activators [64]. WRKY TFs have also been reported to upregulate ABA-responsive genes like* ABF4*,* ABI4*,* MYB2*,* Dehydration Response Element Binding Protein 1a *(*DREBP1a*),* DREBP2a*, and* Response to ABA 18* (*RAB18*). Several WRKY TFs have been reported to be positive regulators of ABA-mediated stomatal closure, while some are negative regulators of seed germination and also indirectly control flowering [65]. In ABA-treated, salt-tolerant rice variety Pokkali, the expression of* WRKY71* increased, while feeble induction was reported for* WRKY24* [66]. Recent findings showed that the expression of WRKY8 was downregulated by the crucifer-infecting tobacco mosaic virus (TMV-cg). In the systemically infected leaves of the* wrky8* mutants, the expression of ABI4 was reduced, while that of 1-aminocyclopropane-1-carboxylic acid synthase 6 (ACS6) and the ethylene response factor 104 (ERF104) was enhanced. The accumulation of TMV-cg was reduced on exogenous application of ABA [67]. WRKY20 isolated from* Glycine soja* was characterized to regulate ABA signaling and enhance drought tolerance. The* GsWRKY20* has also been reported to be associated with flowering with high expression in the shoot tips and the inflorescence meristems of wild soybean [68].

## 8. Transgenic Approaches for Overexpression of WRKY Proteins during Abiotic Stress

### 8.1. Drought and Salinity Stresses

**Figure 2:**
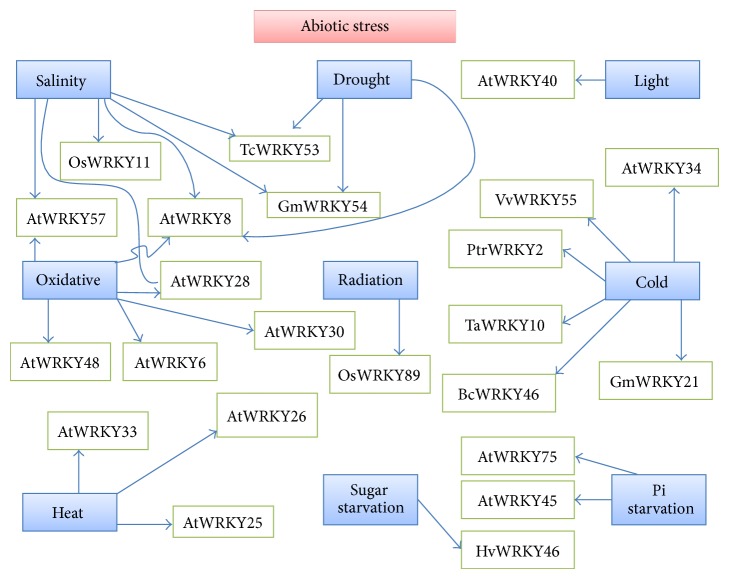
WRKY proteins regulating plant responses against multiple abiotic stresses like salinity, drought, heat, cold, nutrient starvation, light, radiation, and oxidative stresses. “At” refers to* Arabidopsis thaliana*, “Os” refers to* Oryza sativa*, “Gm” refers to* Glycine max*, “Vv” refers to* Vitis vinifera*, “Hv” refers to* Hordeum vulgare*, “Ta” refers to* Triticum aestivum*, “Bc” refers to* Brassica campestris*, and “Ptr” refers to* Poncirus trifoliata*.

Prolonged drought in a particular area results in a physically dry soil which is unsuitable for crop productivity. On the other hand, high salt concentration gives rise to a physiologically dry soil which is also antagonistic for sustainability of the crops. Thus, drought and salinity stresses merge at a common point of water shortage, thus inhibiting crop productivity. This is the reason for which most of the WRKY protein-mediated responses for both stresses are very common in nature. Since the response is overlapping, it becomes difficult to divide the WRKY proteins into a particular group responsible for drought tolerance and another responsible for salt tolerance [69]. Under the control of* HSP101* promoter, the overexpression of* OsWRKY11* resulted in lower rates of leaf-wilting and enhanced chlorophyll stability, along with sustenance of the green parts. These factors help in generating higher drought tolerance in the crop [70] (Figure 2). The 35S : OsWRKY45 and 35S : OsWRKY72* Arabidopsis* plants had higher expression of the ABA-inducible genes which aided in developing higher salt and drought tolerance in the plant [71]. Overexpression of* GmWRKY54* from* Glycine max* in transgenic lines enhanced the salt and drought tolerance (Figure 2). High levels of GmWRKY54 are thought to manipulate the expression of the TF gene,* salt tolerance Zn finger* (*STZ/Zat10*), and* DREB2A*. The transgenic plants overexpressing the GmWRKY13 had decreased sensitivity to ABA, while these plants had less tolerance towards high salt and mannitol in comparison to the wild types. Thus, it can be inferred that GmWRKY13 is a negative regulator of abiotic stress response when overexpressed alone [72]. Exposure to salt and drought stress induced the expression of* TcWRKY53* in* Thlaspi caerulescens*. Two ethylene response factor (ERF) family genes in tobacco,* NtERF5* and* NtEREBP-1*, had low transcription levels in transgenic tobacco plants overexpressing* TcWRKY53* [73]. The expression of the Late Embryogenesis Abundant (LEA) family gene* NtLEA5* remained unaffected, indicating the fact that TcWRKY53 increases osmotic stress tolerance through interaction with an ERF-type TF. It is probable that TcWRKY53 does not directly manipulate the stress-responsive genes [73]. Overexpression of either* AtWRKY25* or* AtWRKY33* conferred salt tolerance in* Arabidopsis*. These two WRKY proteins are closely related and their transcript levels subsequently increased when the plants were treated with high concentrations of salt. The importance of these proteins was proved from a mutation experiment considering* Atwrky33* null mutants and double mutants of* Atwrky25* and* Atwrky33*. Both types of mutants exhibited increased sensitivity to saline stress [34]. Arrest of seed germination under the influence of ABA is levied by AtWRKY2, which has been proposed to act as a negative feedback regulator of ABA-induced seed dormancy [74]. AtWRKY57, AtWRKY8, and AtWRKY28 regulate signaling cascades in salinity, drought, osmotic, and oxidative stresses [75] (Figure 2).

Results furnished by suitable experiments showed that eight out of 15* WRKY* genes in wheat are induced by high salt concentrations, PEG, cold, or heat [29]. Overexpression of OsWRKY30 activated by a MAPK cascade enhanced the tolerance of transgenic rice to drought [40]. The salt and drought tolerance dramatically increased when* TaWRKY10* from wheat was introduced and overexpressed in tobacco. TaWRKY10 has been depicted as a major TF, activating multiple stress-related genes, and also has a role in maintaining the osmotic balance in the cell. The transgenic tobacco lines exhibited remarkably high levels of proline and soluble sugar but low levels of malondialdehyde (MDA) when exposed to drought or salt stress [76].* TaWRKY2* and* TaWRKY19* overexpression in* Arabidopsis* led to more efficient salt, drought, and low temperature tolerance. The overexpression of* BcWRKY46* and* HvWRKY38* in* Arabidopsis* resulted in enhanced tolerance towards drought and salt stresses [76]. The protein products of these genes are nuclear proteins and act as TFs, targeting many downstream genes which need to be activated to combat abiotic stress [77]. Exposure of* Arabidopsis* to salinity stress resulted in twofold increase in the levels of 18* WRKY* transcripts and repression of eight* WRKY* genes [58]. We have already discussed the induction of ABA-responsive genes due to the AtWRKY40-ABAR (ABA-binding protein) interaction. AtWRKY18 and AtWRKY60 act in synchronization to increase the sensitivity of the plant towards salt and osmotic stresses [14]. The expression of* PtrWRKY2* gene in* Poncirus trifoliata* was suppressed by 27–50% upon exposure to prolonged drought stress. However, in drought-stressed* Citrus maxima* (pummelo) plants, the expression pattern of* PtrWRKY2* remained unaltered [78]. Salt and ABA treatment increased the transcript levels of* OsWRKY08*. Osmotic stress tolerance via positive regulation of two ABA-dependent genes like* AtCOR47* and* AtRD21* was reported in transgenic* Arabidopsis* overexpressing* OsWRKY08* [24]. Recent reports highlighted the upregulation of* AtWRKY46* during osmotic stresses like salinity and drought. The roles of* WRKY46* in mediating cellular osmoprotection and redox homeostasis under stress have also been depicted via microarray analysis. Regulation of light-dependent starch metabolism is performed by WRKY46 through the control of the* QUA-QUINE STARCH* (*QQS*) gene expression [79]. The group II family of WRKY TFs (JcWRKY) found in the biofuel crop* Jatropha curcas* developed tolerance against ionic, osmotic, and chemical stresses when expressed in* E. coli*. Transcript analysis showed that transcription of* JcWRKY* was increased in response to salinity, dehydration, salicylic acid, methyl jasmonate, and the collar rot fungus* Macrophomina* [80]. In cotton (*Gossypium hirsutum*), a Group IId* WRKY* gene* GhWRKY17* was found to be associated with salt and drought stress. Increased drought and salt sensitivity resulted in transgenic tobacco plants (*Nicotiana benthamiana*) overexpressing* GhWRKY17*. GhWRKY17 lowered ABA sensitivity leading to low transcription of ABA-inducible genes like* AREB*,* DREB*,* NCED*,* ERD*, and* LEA* [81]. These instances obviously indicate the enormous influence of the WRKY proteins in activating a proper response against salt and dehydration. The plants which appear to be tolerant under these harmful environmental circumstances show persistence of green parts. This has been obviously due to shielding of chlorophyll and other necessary pigments required for photosynthesis. Thus, the WRKY TFs must be playing crucial roles in guarding these pigments against the low water status of the cell and preventing influx of salt beyond the threshold limits. The content of Reactive Oxygen Species (ROS) in the tissues of the tolerant varieties is also low [82, 83]. 61* WRKY* genes were identified in* Populus* which were induced by both abiotic and biotic treatments like infection with* Marssonina brunnea*, SA, methyl jasmonate, wounding, cold, and salinity. 46 genes from this cluster were shown to be expressed in roots, stems, and leaves [84]. These results probably hint towards the role of WRKY TFs as activators of genes encoding LEA proteins like dehydrins or even some genes in the biosynthetic pathways of compatible solutes like proline, polyamines, and so forth.

### 8.2. Oxidative Stress

WRKY has often been depicted as an aggravator of ROS production in cells. The ROS like superoxide, hydroxyl radicals, and hydrogen peroxide have tremendous negative impact on the concerned cell wall leading to lipid peroxidation, cell damage, and oxidative stress. The uptake of oxygen is responsible for such oxidative burst of ROS [82, 85]. Hydrogen peroxide treatment in* Arabidopsis* triggered higher expression of AtWRKY30, AtWRKY75, AtWRKY48, AtWRKY39, AtWRKY6, AtWRKY53, AtWRKY22, and AtWRKY08 [86] (Figure 2). The WRKY proteins also help in quenching the ROS produced in mitochondria. The changes in environmental conditions and retrograde signaling affect the expression of several nuclear genes encoding mitochondrial proteins. About 72 WRKY proteins in* Arabidopsis* genome have been proved to regulate production of nuclear transcripts encoding mitochondrial proteins. These WRKY proteins do have WRKY domains complementary to the W-box at the promoters on the nuclear transcripts [87]. WRKY binds to the promoters of marker genes like* Alternative oxidase1a* (*AOX1a*)*, NADH dehydrogenase B2*, and the* AAA ATPase Ubiquinol-cytochrome c reductase synthesis1*. The effects of antimycin A-induced mitochondrial retrograde expression and high light-induced stress were reduced by the overexpression of* AtWRKY40* [88]. On the contrary, AtWRKY63 acts as an activator in inducing high light stress tolerance. It can be assumed that high light-induced stress leads to the formation of ROS which can be somehow quenched by downstream responses triggered by AtWRKY63. Coordination in the coding of stress-responsive genes in mitochondria and chloroplast has been studied through the functions of AtWRKY40 and AtWRKY63. These proteins regulate the expression of stress-responsive genes common to both mitochondria and chloroplasts without disturbing the constitutive expression of the house-keeping genes in other organelles [87]. Another instance of WRKY proteins involved in oxidative and light stress is the T-DNA knockout mutant of* APX1* gene which led to the induction of* AtWRKY6*,* AtWRKY18*,* AtWRKY25*,* AtWRKY33*,* AtWRKY40*,* AtWRKY46*,* AtWRKY54*,* AtWRKY60*, and* AtWRKY70* [13]. AtWRKY70 exhibited constitutive expression in the* Atapx1* mutants [89]. Thus, WRKY proteins indirectly do help in the scavenging of ROS in order to alleviate oxidative stress.

The key proponents of the ROS signaling cascade are ascorbate peroxidases (APX), NADPH oxidases, and Zn finger proteins. Researchers have proposed the relation between Zn finger proteins and WRKY factors in alleviating ROS toxicity. AtWRKY25 could not be formed in appropriate amounts in* Atzat12* mutant (gene for Zn finger protein) plants exposed to high levels of hydrogen peroxide. Thus, it can be obviously suggested that the expression of* AtWRKY25* is dependent on the protein encoded by* AtZat12* [90]. The TF TaWRKY10 of wheat when overexpressed in transgenic tobacco decreased the accumulation of MDA and lowered the levels of superoxide radical and hydrogen peroxide formation on exposure to salinity and drought stresses. Low MDA was attributed to low rates of lipid peroxidation. The transgenic seedlings showed increased tolerance towards oxidative stress due to higher accumulation of TaWRKY10 [77]. The transgenic tobacco plants overexpressing* GhWRKY17* exhibited higher sensitivity towards oxidative stress. The expression of the genes for ROS-scavenging enzymes like* APX, catalase* (*CAT*), and* Superoxide Dismutase* (*SOD*) was suppressed in the transgenic lines [81]. The* Arabidopsis* lines overexpressing the TFs, Helix Loop Helix17 (HLH17) and WRKY28, showed enhanced tolerance towards osmotic stress [75].* WRKY30* was rapidly expressed as a primary response to hydrogen peroxide and methyl viologen (MV). MV acts as a superoxide anion propagator in light [91]. Transgenic* Arabidopsis* plants overexpressing* WRKY15* were more sensitive to both salinity and oxidative stresses with the formation of increased leaf area and accumulation of increased plant biomass. WRKY15 induced leaf area increment through intensified endoreplication and not by extending the cell numbers.* WRKY15* expressed under oxidative stress aids in the activation of mitochondrial genes which code for proteins belonging to the family of mitochondrial dysfunction regulon. WRKY15 has also been proposed as a general repressor of genes whose products participate in mitochondrial retrograde signaling [92]. During dark-induced senescence, the leaves of* Pelargonium* cuttings showed high accumulation of WRKY6. The basis of an increment in ROS level together with higher expression of senescence-associated protease homologs* PeSAG12-1* and* PeWRKY6-1* could not be explained [93]. OsWRKY42 has been portrayed as a negative regulator in oxidative stress. This is because overexpression of* OsWRKY42* in rice resulted in high accumulation of ROS. It was also reported that* OsWRKY42* binds to the W-box of the* OsMT1d* (rice* Metallothionein 1d*) gene and represses its expression, thereby promoting leaf senescence [94].* TaWRKY10* gene isolated from* Triticum aestivum* was reported to be a positive mediator of plant tolerance against salinity and drought through the regulation of osmotic balance, ROS scavenging, and transcription of stress-related genes [95].

Osmotic and oxidative stresses are linked at a point as both decrease viability of crops in general. This cross talk in the signaling cascades of the plant in developing tolerance against varied abiotic stresses has obviously opened several new avenues of research. One of them is to genetically modify some particular target components in the plant system in order to design a transgenic crop which can be tolerant to multiple abiotic stresses. Our discussion gives a clear indication of the fact that WRKY proteins are emerging players in generating abiotic stress tolerance in crops. Though this group of TFs are more related to biotic stresses like pathogen attack and plant defence, their immensely growing significance in response to abiotic stress cannot be neglected at all.

### 8.3. Temperature Stress

Physiological processes in a plant system are best operated at an optimum temperature. Most of these pathways are dependent on enzymes, which have maximum activity at an optimum temperature. Beyond this limit, the activity of the enzymes gradually decreases as the protein structures are affected and ultimately the entire pathway comes to a halt. This is true for temperatures which are either higher or lower than the optimum operating temperature of the system. Several researches have been undertaken to study the growth patterns in crops exposed to extremes of temperature. This has revealed the important roles played by WRKY proteins in response to such stresses.

Tolerance to heat stress has been depicted to be regulated by the Group I WRKY proteins, AtWRKY25, AtWRKY26, and AtWRKY33 (Figure 2). When the* Arabidopsis* plant is exposed to high temperature stress, the expression of* AtWRKY33* is repressed, while that of* AtWRKY25* and* AtWRKY26* is stimulated. Inhibited seed germination, lower survival, and electrolytic leakage were seen in the heat-stressed plants having mutations at the above three loci. On the contrary, increased tolerance towards heat stress was recorded in transgenics overexpressing* AtWRKY25, AtWRKY26,* and* AtWRKY33* [34]. The fact that these genes are activated by a heat-induced ethylene-dependent response has raised the probability of possible convergence and cross talk between the ethylene signaling pathways and the signaling cascades activated in response to heat stress [34]. A possible cross talk between biotic and abiotic stress response components has also been noted in case of AtWRKY39. The* Arabidopsis* gene* AtWRKY39* is induced in response to heat stress and the WRKY protein encoded by this gene positively regulated the interaction between the SA and JA signaling cascades [96]. Heat stress has been found to regulate about nine of the 60 analyzed* WRKY* genes. Out of these nine genes,* AtWRKY7* has been identified as a Heat Shock Factor A1a/1b (HsfA1a/1b), triggering the heat stress-induced genes [97]. We have previously discussed that overexpression of* OsWRKY11* under the control of* HSP101* promoter led to increased drought tolerance. It was reported that these transgenic plants evolved tolerance towards high temperature stress as well [70].

*AtWRKY34* expression is stimulated in response to pollen-specific cold stress.* AtWRKY34* has been proved to be a crucial locus in cold stress regulation as overexpression of* AtWRKY34* makes the pollens sterile even under normal growth conditions [98]. The mutation of this gene enhances the cold stress tolerance in the pollens. Thus, AtWRKY34 negatively regulates the development of cold stress tolerance in pollens. This regulation is achieved by proper manipulation of the transcriptional activators, namely, C-repeat Binding Factors (CBFs) [99]. Increased tolerance to cold stress has also been reported in transgenic* Arabidopsis* overexpressing* GmWRKY21* [65]. Transgenic plants overexpressing* TaWRKY10* also showed enhanced cold stress tolerance [77]. The* Poncirus trifoliata WRKY* gene* PtrWRKY2* is an important player in cold stress (Figure 2). The expression of this gene increased initially when both cold-tolerant* Poncirus* and cold-sensitive pummelo were exposed to cold stress. However, the gene expression subsided in both cold-tolerant* Poncirus* and cold-sensitive pummelo after exposure to 1 hour and 1 day of cold stress, respectively [78]. HvWRKY38 has a role in freezing tolerance as the expression of the corresponding gene was strongly stimulated when the plants were constantly subjected to freezing temperatures [99]. Similarly, PtrWRKY2 may also have some role in developing freezing tolerance in* Poncirus* or pummelo. In rice, 41 out of 103* WRKY* genes exhibited variable expression patterns in response to chilling stress [58]. 25* WRKY* genes in* Glycine max* showed differential transcription patterns in response to cold [72]. In* Vitis vinifera* (grapevine), majority of the 59* VvWRKY* genes were expressed in tissues of young and mature leaves, tendrils, stem apex, roots, and young and ripened fruits [100]. The gene-chip based data was analysed and it was reported that 36* VvWRKY* genes had their expressions increased by twofold on exposure to cold. Phylogenetic studies have confirmed the possibility of a cross talk between stress responses to salt, PEG, and cold-dependent VvWRKYs. The* VpWRKY3* is a homologous gene of* VvWRKY55*. On exposure to cold, the levels of VpWRKY3 steadily increased in the cell, while* VvWRKY55* expression was upregulated under extremely low temperatures [100]. The expression of* VvWRKY43* increased in* Solanum dulcamara* in the colder months [101]. In the roots of barley exposed to cold, transient increase in the expression of* WRKY38* occurred. Cold stressed Pak-choi had their* BcWRKY46* genes upregulated. Transgenic tobacco plants which exhibited constitutive expression of BcWRKY46 were more tolerant to cold stress in comparison to the wild type plants [102]. The expression of WRKY71 in banana peaked when the plant was treated under extremely low temperature conditions [103]. Antagonistic effects in relation to abiotic and biotic stresses were seen in rice plants with overexpressed* OsWRKY76*. In such plants, a specific set of* Pathogenesis related (PR)* genes and other genes involved in phytoalexin synthesis, after inoculation with blast fungus, were downregulated, while the cold stress-associated genes like peroxidase and lipid metabolism genes were upregulated [104].

### 8.4. Stress due to Deficiency of Nutrients

Since plants are sessile organisms, they are completely dependent on the nutrients and essential elements present in their rhizosphere. Deficiency in an important element is strongly antagonistic to proper plant development. This also leads to impairment of multiple physiological pathways as they are always linked at some level or through a common intermediate or cofactor. Salinity or drought is actually a condition related with the characteristics of soil components. In salinity, the NaCl content of the soil surpasses the threshold level to sustain plant growth, while, in drought, the moisture content of the soil is not enough to support germination or development. Nutrient deficiency also falls in the same brackets as salinity and drought. This is because nutrient deficiency is also soil-related.

WRKY TFs are major regulators in overcoming the adversities related to nutrient deficiency in plants grown on nutrient deficient soil (Figure 2). The first WRKY protein involved in nutrient deficiency was AtWRKY75. The gene encoding AtWRKY75 exhibited stimulated expression under phosphate (Pi) deficient conditions. Mutations resulting in silencing of* AtWRKY75* showed higher plant sensitivity towards Pi stress accompanied with reduced absorption of Pi. The* AtWRKY75* RNAi plants had reduced expression of Pi starvation-induced genes like phosphatases, Mt4/TPS1 like genes, and transporters with high affinity for transporting Pi [105]. A negative regulator of* Phosphate 1* (*PHO1*) expression in* Arabidopsis* is the AtWRKY6. The transgenic plants overexpressing AtWRKY6 were more susceptible to Pi deficiency and had a similar phenotype as the* Atpho1* mutants. This led the researchers to assume the control of AtWRKY6 in the expression of* PHO1* and this interaction was indeed proved via ChIP-qPCR analysis. The two W-boxes adjoining the* AtPHO1* promoter are responsible for proper binding of AtWRKY6 through its WRKY domain [106]. It has been suggested that AtWRKY75 and AtWRKY6 differentially and cooperatively regulate the responses to Pi deficiency. AtWRKY6 also acts as a positive mediator of plant responses during boron deficiency [107, 108]. AtWRKY45 is mainly localized in the nucleus. The concentration of AtWRKY45 peaked in the roots typically facing Pi starvation.* AtWRKY45* RNAi plants showed decreased uptake and hence lower accumulation of Pi during Pi starvation when compared to the wild type plants. The AtWRKY45 RNAi plants were also very sensitive to arsenate present in the soil. The expression of* Phosphate Transporter 1;1 *(*PHT1;1*) was stimulated in the transgenic lines overexpressing* AtWRKY45*. These results have depicted AtWRKY45 as a positive regulator in survival against Pi deficiency. An epistatic genetic regulation between AtWRKY45 and PTH1;1 was also reported [109, 110].

Apart from positively regulating the responses to combat Pi starvation,* WRKY45* and* WRKY65* are also expressed during carbon starvation. Thus, the WRKY proteins encoded by these genes act as TFs to upregulate the expression of downstream stress-responsive genes [107, 108]. Increased sensitivity to sugar starvation was reported in a transgenic* Arabidopsis* line overexpressing WRKY72 [71]. The* Arabidopsis Nucleoside Diphosphate Kinase 3a* (*NDPK3a*) expression is stimulated in presence of sugar and the protein encoded by the gene gets localized in the mitochondria. The mitochondrion is the chief reservoir of ATP produced mainly by the oxidation of sugars. The* NDPK3a* promoter has two W-boxes which aid in the binding of SUSIBA2 (HvWRKY46) in barley plants [109, 110]. There are also reports of AtWRKY4 and AtWRKY34 in mediating the expression of* NDPK3a*. SUSIBA2 also regulates the expression of* ISO1* and* Sugar Response Element IIb* (*SREIIb)* in sugar signaling [109, 110]. Further researches and critical analyses are yet to be made on the queer role of WRKY proteins connecting the plant responses to tackle Pi and sugar starvation. This link may be an indication towards a beneficial symbiotic interaction between relative availability of Pi in the soil and sugar metabolism [109, 110].

### 8.5. Radiation Stress

The least studied among all the abiotic stress is the radiation stress and its associated effects on the plant system. However, this field is also gradually gaining enormous importance due to uneven distribution of sunlight throughout the globe. Indiscriminate uses of chlorofluorocarbons (CFCs) and other toxic chemical compounds have led to the formation of the ozone hole. The ozone layer which once acted as an absolute absorber of UV radiations is gradually losing its efficiency. Thus, the plants growing in areas falling under such ozone holes are more prone to UV stress. This is where the significance of designing plants resistant to radiation stress lies.

Radiation stress mainly induces the bleaching of photosynthetic pigments and generation of ROS. Thus, the* WRKY* genes which encode proteins involved in scavenging of ROS obviously get upregulated. We have already discussed these genes which regulate oxidative stress in plants. However, a WRKY gene* OsWRKY89* was identified in rice which has been depicted to regulate responses during UV-B stress (Figure 2). Increased wax deposition on the surfaces of leaves was reported in UV-B stressed transgenic plants overexpressing* OsWRKY89* [111]. The increased wax deposition drastically reduced the percentage of UV-B transmittance through the leaves. Further researches are required to create a database on the involvement of multiple WRKY proteins regulating the responses induced by radiation stress.

## 9. Conclusion

**Figure 3:**
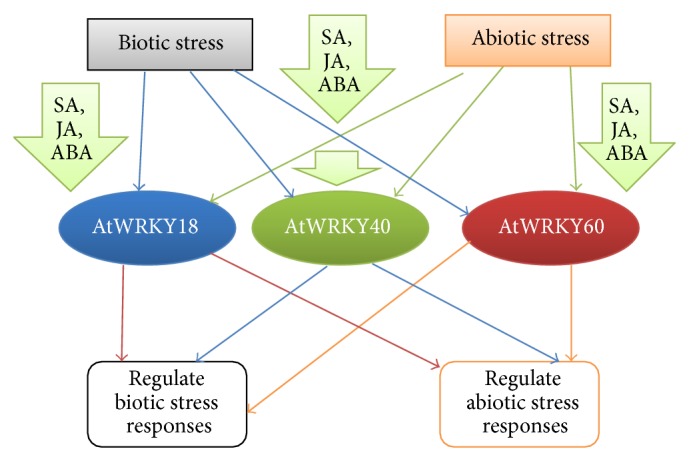
Specific WRKY proteins like AtWRKY18, AtWRKY40, and AtWRKY60 have been depicted as mediators of cross talk between plant responses against abiotic and biotic stresses. It has been reported that these proteins get accumulated in response to SA and JA during biotic stress as well as ABA during abiotic stress responses.

In this review, we emphasized the most recent advances on WRKY proteins. WRKY proteins have been thought to have more significance in regulating biotic stress response as compared to abiotic stress. However, modern research works and trailblazing experiments have proved their pivotal roles for developing plant tolerance towards abiotic stress. WRKY proteins have several interacting partners which together coordinate multiple signal cascades. The relation between MAPKs and WRKY proteins also indicates a cooperative signal transduction cascade. The ABA-inducible genes involved in abiotic stress responses are also dependent on their activation by MAPKs or sometimes Calcium Dependent Protein Kinases (CDPKs). A potential cross talk between the MAPKs activating WRKY TFs and those upregulating the abiotic stress response gene is yet to be reported. Improved technologies along with molecular, computational, and informational agrobiology may furnish further details in this respect in near future. Future prospects in this field also include the possibility of a cross talk between abiotic and biotic stress responses mediated by WRKY factors. The structurally related proteins AtWRKY18, AtWRKY40, and AtWRKY60 have provided some hint towards the occurrence of such cross talk (Figure 3). This is because these proteins are participants in the pathways regulated by the three major phytohormones of plant system, that is, SA, JA, and ABA [14]. While SA and JA are involved in transducing response against biotic stress, ABA is an essential proponent in the abiotic stress pathways. WRKY18, WRKY40, and WRKY60 also act as an activator of the* IaaH* (*indole-3-acetamide hydrolase*) and* IaaM* (*tryptophan monooxygenase*) genes of the T-DNA after its integration in the plant following* Agrobacterium* infection. Thus, WRKYs play a role in the expression of the oncogenes and inducing crown gall disease [112]. In* Populus tomentosa*, overexpression of Group IIa* WRKY* gene,* PtoWRKY60*, resulted in the upregulation of the* PR5.1*,* PR5.2*,* PR5.4*,* PR5.5*, and other defense-associated genes [113]. Double mutants of* WRKY18* and* WRKY40* enhanced* Arabidopsis* resistance against powdery mildew fungus,* Golovinomyces orontii* by transcriptional reprogramming, alterations in the SA or JA signalling, and* Enhanced Disease Susceptibility1* (*EDS1*) expression, along with accumulation of camalexin. It was further hypothesised that this fungus required the two WRKY proteins for successful infection [114, 115]. Another instance of a cross talk between abiotic and biotic stress responses is the function of AtWRKY25 and AtWRKY33. These proteins show accumulation both under abiotic and biotic stresses. Abiotic stresses like NaCl and high temperature or pathogenic invasion by* Pseudomonas syringae* induced the expression of* AtWRKY25* and* AtWRKY33* [116]. Future prospects also involve the possible identification of specific WRKY proteins which can develop plant tolerance against multiple abiotic stresses. An example of a WRKY protein in this context is the AtWRKY8 which upregulates the plant tolerance against salinity, drought, and also oxidative stress (Figure 2). Genetic engineers can target such single WRKY factors and design multistress tolerant transgenic plant lines. Such tolerant lines can be designed in cereal food crops like rice. Seeds of such crops can be distributed to farmers across wide geographical areas for compatible growth even under harsh environmental conditions.

## Figures and Tables

**Table 1:** Major families of WRKY proteins with associated characteristics.

Gene family name	Gene family subclass	Protein associated	Protein type	References
Group I	—	AtWRKY1, AtWRKY2, AtWRKY1, AtWRKY2, AtWRKY3, AtWRKY4, AtWRKY10, AtWRKY19, AtWRKY20, AtWRKY25, AtWRKY26, AtWRKY32, AtWRKY33, AtWRKY34, AtWRKY44, AtWRKY45, AtWRKY58, AtWRKY73, OsWRKY24, OSWRKY70, OsWRKY78, OsWRKY30, OsWRKY4, OsWRKY63, OsWRKY61, OsWRKY81	The proteins contain two WRKY domains	
Group II (*Arabidopsis*)	IIa	AtWRKY18, AtWRKY40, AtWRKY60	Single WRKY domain with the same Cys2-His2 Zn finger motif	
	IIb	AtWRKY6, AtWRKY9, AtWRKY31, AtWRKY36, AtWRKY42, AtWRKY47, AtWRKY61, AtWRKY72		
	IIc	AtWRKY8, AtWRKY12, AtWRKY13, AtWRKY23, AtWRKY24, AtWRKY28, AtWRKY43, AtWRKY48, AtWRKY49, AtWRKY50, AtWRKY51, AtWRKY56, AtWRKY57, AtWRKY59, AtWRKY68, AtWRKY71, AtWRKY75		
	IId	AtWRKY7, AtWRKY11, AtWRKY15, AtWRKY17, AtWRKY21, AtWRKY39, AtWRKY74		[13, 14, 20–22, 117, 118]
	IIe	AtWRKY14, AtWRKY16, AtWRKY22, AtWRKY27, AtWRKY29, AtWRKY35, AtWRKY65, AtWRKY69		
	IIIa	AtWRKY38, AtWRKY62, AtWRKY63, AtWRKY64, AtWRKY66, AtWRKY67		
Group II (rice)	—	OsWRKY51, OsWRKY42, OsWRKY25, OsWRKY44, OsWRKY68, OsWRKY6, OsWRKY37, OsWRKY66, OsWRKY2, OsWRKY13		
Group III (*Arabidopsis*)	IIIb	AtWRKY30, AtWRKY41, AtWRKY46, AtWRKY53, AtWRKY54, AtWRKY55, AtWRKY70	Single WRKY domain with different Cys2-His/Cys Cys2-His2 Zn finger motif	
Group III (rice)	—	OsWRKY22, OsWRKY20, OsWRKY69, OsWRKY74, OsWRKY15, OsWRKY19, OsWRKY45, OsWRKY75		
Group IV	IVa	OsWRKY33, OsWRKY38	Presence of partial Zn finger motif	
	IVb	VvWRKY02, VvWRKY29, OsWRKY56, OsWRKY58, OsWRKY52	Loss of Zn finger motif	

“At” refers to *Arabidopsis thaliana*, “Os” to *Oryza sativa,* and “Vv” to *Vitis vinifera*.

**Table 2:** Role of WRKY proteins in both abiotic and biotic stresses.

Stress type	Gene	Inducible factors	Function in stress	References
Abiotic stress	AtWRKY2	NaCl, mannitol	Negatively regulates ABA signaling	[74]
	AtWRKY18	ABA	ABA signaling and salt tolerance	[119]
	AtWRKY26	Heat	Heat tolerance	[34]
	AtWRKY39	Heat	Heat tolerance	[96]
	AtWRKY40	ABA	ABA signaling	[119]
	OsWRKY08	Drought, salinity, ABA, and oxidative stress	Tolerance towards oxidative stress	[120]
	OsWRKY11	Heat, drought	Xerothermic stress tolerance	[121]
	OsWRKY89	Salinity, ABA, and UV-B	UV-B radiation tolerance	[122]
	OsWRKY45	Salt, drought	Salt and drought tolerance	[123]
	OsWRKY72	Salt, drought	Salt and drought tolerance	[124]
	GmWRKY21	Salt, cold, and drought	Cold tolerance	[72]
	GmWRKY54	Salt, drought	Salt and drought tolerance	[72]

Biotic stress	AtWRKY38	Target of NPR1 during Systemic Acquired Resistance (SAR)	Increases Salicylic Acid- (SA-) mediated response	[125]
	AtWRKY53	Target of NPR1 during SAR	Increases SA- mediated response	[125]
	AtWRKY66	Target of NPR1 during SAR	Increases SA- mediated response	[125]
	AtWRKY70	Target of NPR1 during SAR	Node of convergence for SA-mediated and jasmonic acid- (JA-) mediated defence signaling	[126]
	OsWRKY31	*Magnaporthe oryzae *	Increased resistance	[127]
	OsWRKY45	*M. oryzae *	Increased resistance	[128]
	OsWRKY77	*Pseudomonas syringae *	Positively regulates plant basal resistance	[129]
	HvWRKY10	Effector Triggered Immunity (ETI)	ETI activator	[130]

“At” refers to *Arabidopsis thaliana*, “Os” to *Oryza sativa*, “Gm” to *Glycine max,* and “Hv” to *Hordeum vulgare*.
